# Genetic Basis for Developmental Homeostasis of Germline Stem Cell Niche Number: A Network of Tramtrack-Group Nuclear BTB Factors

**DOI:** 10.1371/journal.pone.0049958

**Published:** 2012-11-21

**Authors:** Mathieu Bartoletti, Thomas Rubin, Fabienne Chalvet, Sophie Netter, Nicolas Dos Santos, Emilie Poisot, Mélanie Paces-Fessy, Delphine Cumenal, Frédérique Peronnet, Anne-Marie Pret, Laurent Théodore

**Affiliations:** 1 Centre de Génétique Moléculaire, Unité Propre de Recherche 3404 du Centre National de la Recherche Scientifique, Gif-sur-Yvette, France; 2 Departement de Biologie, University of Versailles St-Quentin, Versailles, France; 3 Laboratoire de Génétique et Biologie Cellulaire, Equipe Associée 4589, University of Versailles St-Quentin, Versailles, France; 4 Departement de Biologie, University of Paris-Sud, Orsay, France; 5 Biologie du Développement Unité Mixte de Recherche 7622, Centre National de la Recherche Scientifique et Université Pierre et Marie Curie, Paris, France; University of Bern, Switzerland

## Abstract

The potential to produce new cells during adult life depends on the number of stem cell niches and the capacity of stem cells to divide, and is therefore under the control of programs ensuring developmental homeostasis. However, it remains generally unknown how the number of stem cell niches is controlled. In the insect ovary, each germline stem cell (GSC) niche is embedded in a functional unit called an ovariole. The number of ovarioles, and thus the number of GSC niches, varies widely among species. In *Drosophila*, morphogenesis of ovarioles starts in larvae with the formation of terminal filaments (TFs), each made of 8–10 cells that pile up and sort in stacks. TFs constitute organizers of individual germline stem cell niches during larval and early pupal development. In the *Drosophila melanogaster* subgroup, the number of ovarioles varies interspecifically from 8 to 20. Here we show that *pipsqueak*, *Trithorax-like*, *batman* and the *bric-à-brac* (*bab*) locus, all encoding nuclear BTB/POZ factors of the Tramtrack Group, are involved in limiting the number of ovarioles in *D*. *melanogaster*. At least two different processes are differentially perturbed by reducing the function of these genes. We found that when the *bab* dose is reduced, sorting of TF cells into TFs was affected such that each TF contains fewer cells and more TFs are formed. In contrast, *psq* mutants exhibited a greater number of TF cells per ovary, with a normal number of cells per TF, thereby leading to formation of more TFs per ovary than in the wild type. Our results indicate that two parallel genetic pathways under the control of a network of nuclear BTB factors are combined in order to negatively control the number of germline stem cell niches.

## Introduction

Compound or multiple organs such as the kidney, liver, and lung in mammals, the vibrissae field in rodents or the eye in insects, are made of functional units that are built under the same reiterated scheme. During morphogenesis of such organs, the final structure must be controlled at the level of the total size of the organ, the total number of units, and the construction of single units. The *Drosophila melanogaster* ovary is a compound organ, made of approximately 20 functional units called ovarioles [1]. At the anterior tip of each ovariole is the terminal filament (TF), contiguous to the germarium that contains both germline and somatic stem cells and produces egg chambers. Ovariole number is defined during larval development by the number of TFs, each constituting an organizing center for the formation of individual ovarioles ([2], for review see [3]).

The *Drosophila melanogaster* female gonad forms at the end of embryogenesis from the apposition of germline cells and somatic gonadal precursors (SGPs) that originate from the posterior mesoderm [4]. Female identity of this organ is then determined by the sex determination cascade [5]. Somatic and germline cells proliferate until early/mid third instar larval stage [6]. Ovariole morphogenesis starts in mid-L3 in the anterior hemisphere of the larval ovary with the recruitment, intercalation and sorting of post-mitotic TF cells (TFCs) [6], [7], [8] implicating the actin-depolymerizing factor Cofilin [9]. This process is reiterated across the ovary from the medial to the lateral side during the second half of the L3 stage, thus constituting a morphogenetic wave. At the onset of the larval-pupal transition, all of the approximately 20 TFs have formed, each TF composed of a stack of 8–10 disc-shaped cells. TFs are the first elements of the niches that will host germline stem cells. However, formation of individual ovarioles does not require the presence of germline cells, as inferred from the correct morphogenesis of ovarioles in *germ cell less^D^* females that are deprived of germline cells [1]. At the late L3 stage, a subset of somatic cells located immediately posterior to TF cells, *i.e.* the cap cells (CCs), are incorporated into the forming germarium [10] and recruit adjacent primordial germline cells to become germline stem cells (GSC, reviewed in [11]). The specification of CCs relies on the activation of the Notch pathway in somatic cells [12], [13], [14]. At the end of the larval stage and in early pupae, apical somatic cells, located directly anterior to the TFs, migrate posteriorly between TF stacks and form an epithelial sheath that surrounds each TF, thereby packaging individual germaria and ultimately separating ovarioles [1]. The formation of TFs appears thus to be the first step in initiating the morphogenesis of ovarioles. TFC and CC together with escort cells (ECs, [15], [16], [17], [18], [19]) form the GSC niches that maintain the GSC state in the adult [20], [21], [22].

Ovariole number is species-specific and varies from *ca*. 8 to 20 between different species of the *melanogaster* subgroup [2], [23], [24], [25], [26]. In *Drosophila melanogaster*, ovariole number depends on environmental conditions, such as temperature, rearing density and nutrition [2], [27], [28], [29], [30]. Several studies have identified intra- and inter-specific Quantitative Trait Loci (QTL) controlling ovariole number (reviewed in [3]), but the genes underlying these QTL remain to be identified [30], [31], [32], [33]. A recent study indicates that in *D. melanogaster*, increasing TFC number by reducing Hippo pathway activity, or increasing both TFC size and number by expressing constitutively active S6K in ovarian somatic cells leads to an increase in TF number and consequently to more ovarioles [34]. Lower ovariole number in *Drosophila yakuba* compared to *Drosophila melanogaster*, as well as that induced by starvation are due to a lower overall number of TFCs per ovary without modification of the number of TFCs stacked per TF. In contrast, higher ovariole number induced by increasing the temperature is caused by reduction in the number of TFCs per TF, without changes in total TFC number or TFC size [34]. Ovariole number can thus be regulated by two different processes: the determination of the total number of TFCs and the sorting of TFCs into TFs during ovariole morphogenesis.

The timing of TF formation is under the control of ecdysone signaling [35], [36]. At the early L3 stage, when ecdysone levels are low, repression of the ecdysone receptor (EcR) target gene *broad* by the EcR/USP (Ultraspiracle) heterodimer is required to repress precocious niche formation and for correct morphogenesis of the ovary [36]. Conversely, at mid-L3, upon ecdysone production, EcR and USP are necessary for niche formation through activation of *broad* expression [36].

Nuclear factors encoded by *engrailed* (*en*) and the *bric-à-brac* (*bab*) locus control TF formation. EN is expressed in forming TFs and in CCs as soon as they are recruited, and maintained in the niche throughout pupal and adult stages [36], [37]. During TF morphogenesis, *en* mutant cell clones integrate into TFs and adopt the flattened shape typical of TFCs, but they do not align properly with wild-type cells [38]. This suggests that *en* may be involved specifically in proper organization and alignment of TFCs into their characteristic stack structure. Strong hypomorphic *bab* alleles display various grades of ovarian defects, including an overall reduction in the size of the ovary, limited oogenesis, and sterility in adults [6], [7], [39], as well as a strong reduction in the number of cells expressing the TF-specific LB27 enhancer-trap reporter in early pupal ovaries [6]. Mutant clonal analysis showed that *bab* mutant TFCs display aberrant features cell autonomously. Similar to *en* mutant cells, *bab* homozygous mutant cells can integrate into TFs but do not align properly with *bab* heterozygous cells. However, *bab* homozygous mutant TFCs tend to retain a rounded shape rather than acquiring the typical flattened shape. It is thus not clear whether *bab* controls the determination of TFCs, or TF morphogenesis during the intercalating process and sorting of TFCs into TF stacks.

The *bab* locus is composed of two related genes, *bab1* and *bab2* [39]. BAB1 and BAB2 proteins share a BTB/POZ domain (Bric-à-brac Tramtrack Broad-complex/Pox viruses and Zinc fingers, thereafter called BTB) [40], [41]. The BTB domain is a highly conserved protein-protein interaction domain of *ca.* 100 amino acids involved in homo- and heterodimer formation [42]. It is found in a protein family with an estimated 40 members in *Drosophila* [40]. BAB proteins interact with each other in the yeast 2-hybrid system and with the Bip2 TATA-box Protein Associated Factor [43]. Based on sequence similarity, it was recently shown that BAB proteins belong to the Tramtrack Group of BTB nuclear factors (ttk group) [44], which also includes Pipsqueak (PSQ) [45], the GAGA factor (GAF) encoded by *Trithorax-like* (*Trl*) [46], and Batman (syn. Lola-like) [47].

In addition to a ttk group BTB domain, BAB and PSQ proteins share the presence of a Pipsqueak domain involved in DNA binding [48]. Despite the similar primary structures of BAB and PSQ proteins, no common phenotype has been described between *bab* and *psq* mutants. In contrast, the three nuclear BTB proteins PSQ, TRL/GAF and Batman were shown to participate to common complexes and to share a common function in the maintenance of Hox gene expression [47], [49], [50], [51]. PSQ binds d(GA)n repeats and is involved in regulation of Hox genes during development [51]. TRL/GAF binds the same d(GA)n repeats than PSQ, however through a DNA-binding domain that is unrelated to the Pipsqueak domain. PSQ is known to interact physically with TRL/GAF through their common BTB domain [49]. PSQ colocalizes broadly with TRL/GAF on polytene chromosomes [49], and TRL/GAF also co-localizes completely with its BTB partner Batman [47], [50]. In addition, the ttk group BTB domains of PSQ, TRL/GAF and Batman have the ability to multimerize [44]. All these data suggest that the three nuclear factors PSQ, TRL/GAF and Batman may act together on many shared target genes. Their relation with factors encoded by the *bab* locus is poorly documented. The BAB1 BTB domain interacts with that of Batman in a 2-hybrid assay in yeast [47] and *batman* and the *bab* locus interact genetically in the control of sex combs on male tarsa [52]. Taken together these results suggest that a group of ttk group BTB factors may constitute a regulatory network regulating common target genes.

Conservation of GA-binding factors among metazoans has long been questioned due to the difficulty encountered in detecting orthologues *in silico* [53]. However, recent studies indicated that d(GA)n repeats are present in mammalian Polycomb Response Elements (PRE) [54], [55] and that a TRL/GAF orthologue, c-Krox/Th-POK, is present in humans [56] and binds Hox gene sequences *in vivo*. These findings support the hypothesis that a conserved nuclear BTB network might be involved in transcriptional regulation of developmental genes in metazoans.

Apart from the partnership between *Trl* and *batman* in Hox gene regulation and activation of pigment cell death in the eye [57], little is known about shared functions of *psq*, *Trl* and *batman* in other developmental processes. Here we report that a striking common feature of a reduction in *psq*, *Trl* and *batman* gene dosage is an increase in the number of ovarioles. We characterized this phenotype and studied the interactions of *psq*, *Trl* and *batman* with the *bab* locus. We show that, whereas the *bab* locus controls the sorting of TFCs into TFs, *psq* plays a major function in global control of TFC number, without affecting sorting of TFCs into TFs.

## Results

### *psq* Negatively Controls the Number of Ovarioles Per Ovary

**Figure 1:**
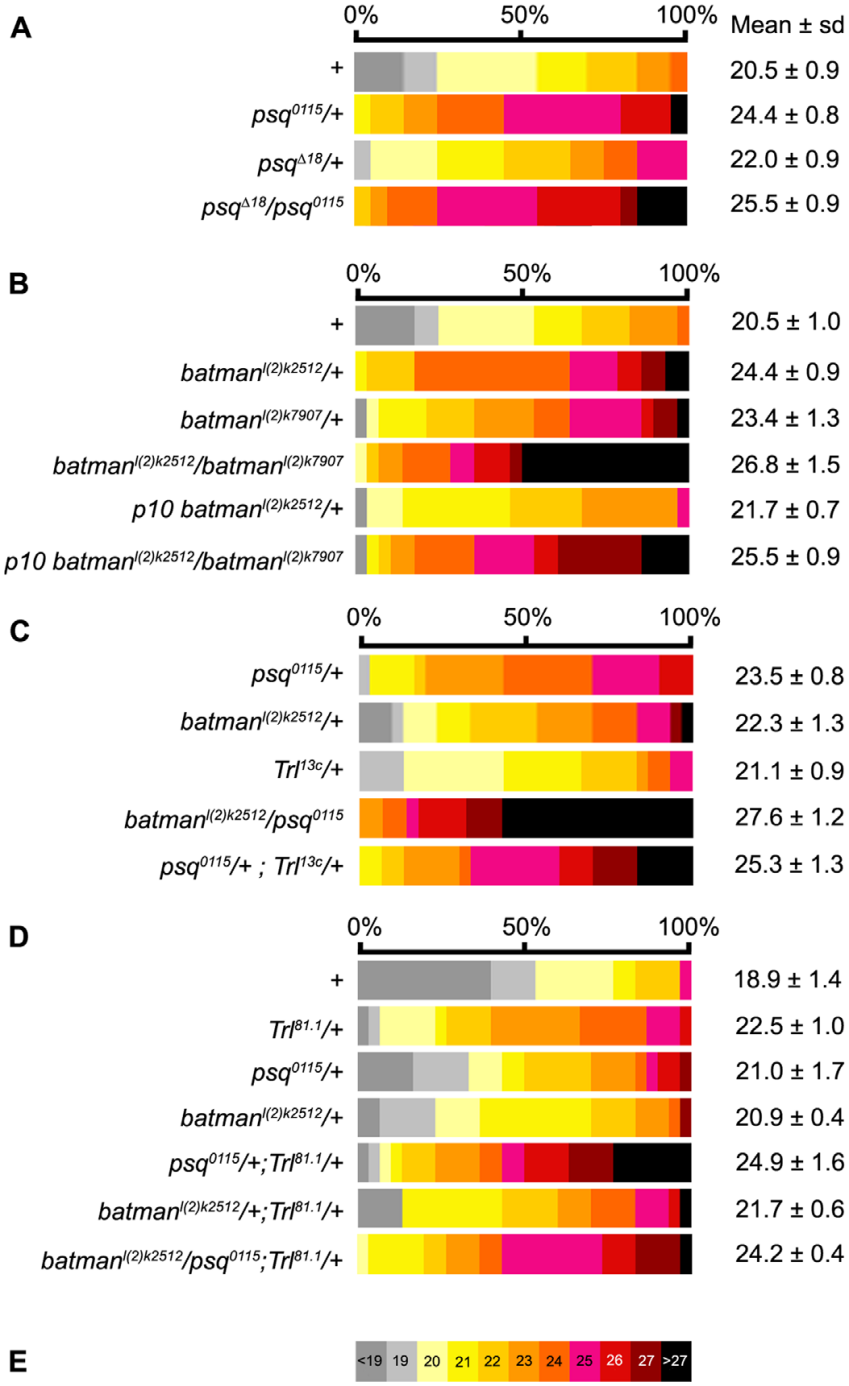
Ovariole number in ttk-BTB group heterozygous combinations. Distribution of the number of ovarioles per ovary in females carrying *psq* (A), *batman* (B) and *Trl* (C) mutant alleles, and in double and triple heterozygotes (C,D). The wild-type control (+) corresponds to Canton-S. Each phenotypic class corresponding to the indicated number of ovarioles per ovary was attributed a color as defined on the color table shown in E. The same color table was used in all representations of ovariole number distribution in this Figure and in Figures 2 and 3. The mean number of ovarioles per ovary is given to the right of each distribution. All values are expressed as the mean +/− sample standard deviation. The sample size was 30 ovaries except for data presented in the A panel for which sample size was 20. All statistics are shown in Table S1.

The *psq* locus is complex and produces several transcripts, encoding two major PSQ isoforms, PSQ-A, that contains a BTB domain and a DNA-binding domain, and PSQ-B, which shares the DNA-binding domain with PSQ-A but lacks the BTB domain [51]. Two previously characterized *psq* alleles were selected, *psq^0115^* that affects the production of both PSQ-A and PSQ-B, and the *psq* ^Δ*18*^ deficiency that affects the production of the PSQ-A protein without affecting that of PSQ-B [51]. The heteroallelic *psq^0115/^psq* ^Δ*18*^ combination was shown to produce sub-viable flies with undetectable levels of the PSQ-A isoform [51]. PSQ-A levels were indeed undetectable as opposed to PSQ-B levels in total protein extracts from *psq^0115/^psq* ^Δ*18*^ ovaries (Figure S1). The global morphology of *psq^0115/^psq* ^Δ*18*^ ovaries from one day-old females was similar to that of Canton-S wild type ovaries from females of the same age. However, *psq^0115/^psq* ^Δ*18*^ females had a dramatic increase in the mean number of ovarioles per ovary (25.5, Figure 1A) when compared to Canton-S females (20.5) grown in the same experimental series (p<0.001, Table S1A), and up to 29 ovarioles per ovary, whereas the maximum for Canton-S was 24 ovarioles per ovary. Balanced stocks of *psq^0115^* and *psq* ^Δ*18*^ mutations were outcrossed to the wild type Canton-S stock and ovariole number was quantified in the heterozygous progeny. The difference in ovariole number (ΔON, Materials and Methods) was normalized using Canton-S flies as a reference (Table S1A’). The average number of ovarioles per ovary increased in *psq^0115/^*+ (24.4, ΔON = 19.0%, p<0.001, Table S1A,A’) and *psq* ^Δ*18*^/+ (22.0, ΔON = 7.3%, p<0.01, Table S1A,A’) when compared to Canton-S. Ovariole number was higher in *psq^0115^* heterozygotes than in *psq* ^Δ*18*^ heterozygotes. This might be due to the fact that *psq^0115^* produces a truncated protein containing the BTB domain that may compete with the wild-type protein, and thereby reduces or poises the function of the wild-type BTB-containing isoforms, as previously suggested [58].

**Figure 2:**
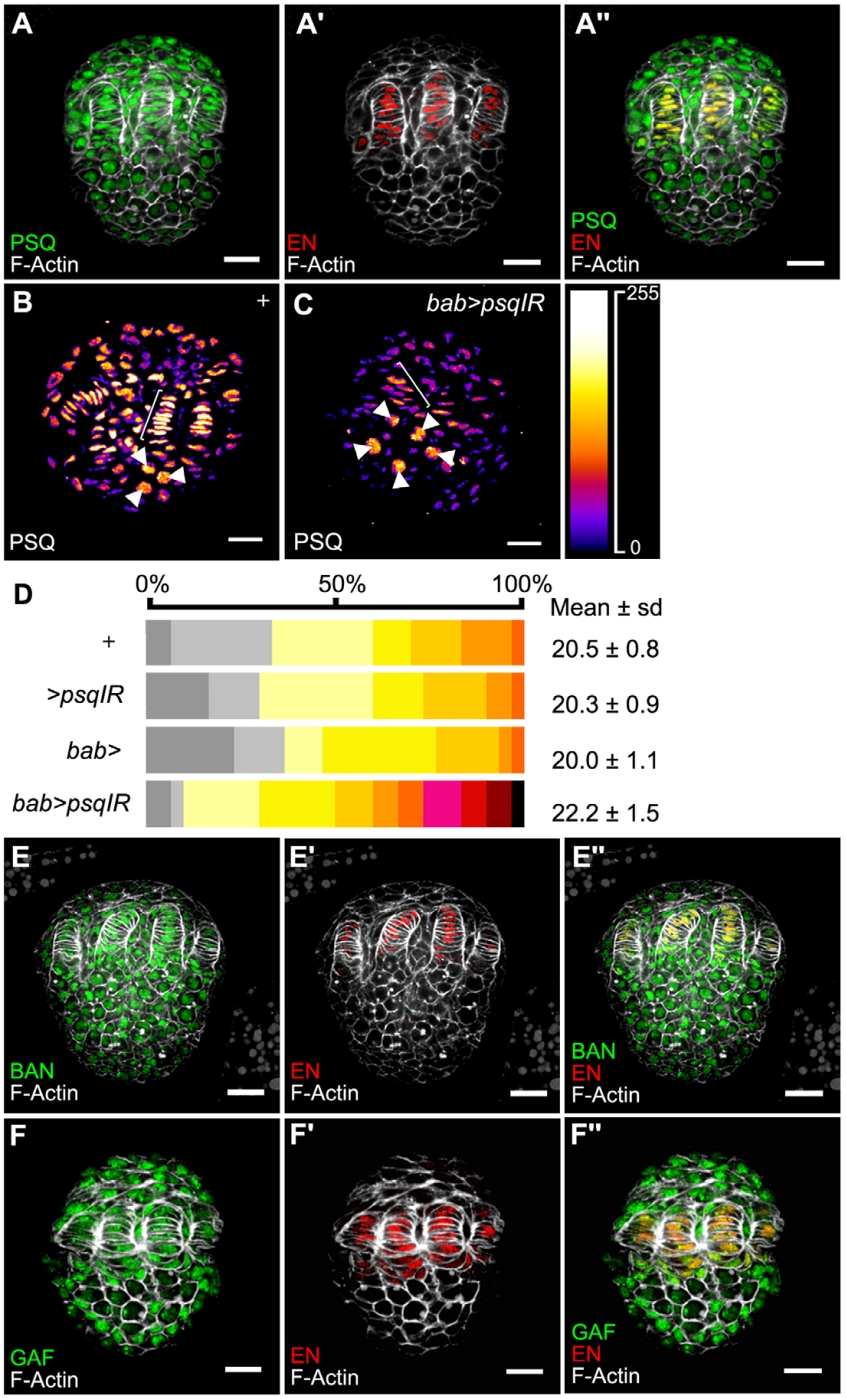
Expression of ttk group proteins in the late third instar larval ovary. (A) Expression of PSQ (green in A, A’’). TFCs are marked with anti-EN (red in A’, A’’) and cell perimeters with F-Actin (white in all images). (B, C) PSQ levels from *bab>psqIR* (*UAS-psqIR/+; bab-Gal4/+*) larval ovaries (C) compared to those of Canton-S (+, B) from the same experimental series. Signal intensity is visualized using the Fire Lookup Table (ImageJ, NIH, inset on the right). The UAS/Gal4 constructs used in this experiment allow expression of Gal4 in somatic cells but not in the germ cells. In *bab>psqIR,* when compared to + ovaries, signal intensity decreases in somatic cells, including TFs (brackets) and remains constant in germ cells (arrowheads). Scale bar: 20 µm. (D) Distribution of the number of ovarioles per ovary in *bab-Gal4* and *UAS-psqIR* combinations. Color table as in Figure 1. The mean number of ovarioles per ovary is given on the right. All values are expressed as the mean +/− sample standard deviation. The sample size was 30 ovaries. All statistics are shown in Table S2A. (E,F) Batman (green, E, E’’) and TRL/GAF (green, F, F’’) expression in L3 ovaries. TFCs are marked with anti-EN (red in E’, E’’, F’, F’’) and cell perimeters with F-Actin (white in all images). Scale bar : 20 µm.

The number of ovariole in both *psq^0115^* and *psq* ^Δ*18*^ heterozygotes was higher than in any wild-type strains analyzed so far. In addition, the effect of *psq* mutations was dominant in a Canton S background, suggesting that the increase in ovariole number is not simply due to various genetic backgrounds from the original *psq* stocks. However, since *psq* ^Δ*18*^ also affects the expression of the adjacent locus *lola* [59], we sought for a way to reduce *psq* function without altering *lola* expression. We took advantage of an available *psq* RNAi line to knockdown *psq* expression using the UAS/Gal4 system (see Materials and Methods). The sequence present in the *psq* RNAi construct includes the exonic sequences encoding the BTB domain, as well as sequences common to all *psq* transcripts. This construct is thus expected to affect expression of all PSQ isoforms. In late L3, when all TFs are already formed, PSQ proteins were detected in the nuclei of all somatic cells, as well as in germline cells (Figure 2A–A’’, B). In order to knockdown *psq* function with RNAi at the larval stage, we used a *bab-Gal4* driver expressed in somatic cells of the larval ovary. This combination allowed strong reduction of PSQ levels in ovarian somatic cells (Figure 2B,C). In this context, the number of ovarioles per ovary in adult females significantly increased (22.2, ΔON = 8,3%, Figure 2D and Table S2B) when compared to that in any of the three control conditions (p<0.01, Table S2A). Taken together, results from mutant analysis as well as RNAi knockdown of *psq* function suggest that the *psq* locus negatively controls the number of ovarioles per ovary. The fact that 1) PSQ-A is the only PSQ isoform that is affected in *psq* ^Δ*18*^ *,* 2) *psq* ^Δ*18*^ heterozygous females have more ovarioles than wild type females, and 3) PSQ-A is undetectable in *psq^0115/^psq* ^Δ*18*^ ovaries (Figure S1) which have about five additional ovarioles when compared to wild type ovaries strongly suggests that this isoform, which contains a ttk group BTB domain, is involved in controlling ovariole number.

### Two other Ttk Group Genes Cooperate with *psq* to Control Ovariole Number

The BTB domain from PSQ is known to interact physically with that of two other ttk group BTB nuclear factors encoded by the *batman* and *Trl* (TRL/GAF) genes [44]. PSQ was previously shown to co-immunoprecipitate from larval salivary gland nuclear extracts with TRL/GAF [49]. TRL/GAF was also shown to co-immunoprecipitate from whole larvae nuclear extracts with Batman [47]. Like *psq*, *Trl* and *batman* are expressed in all somatic and germline cells of the ovary (Figure 2E–F’’). We thus addressed whether *Trl* and *batman* are also involved in regulation of ovariole number. *batman^l(2)k02512^* is a strong hypomorphic allele, whereas *batman^l(2)k07907^* is a weaker allele [47]. The morphology of *batman* mutant ovaries was similar to wild type. The average ovariole number per ovary was significantly higher in *batman^l(2)k07907^*/+ (23.4, ΔON = 14.1%, p<0.001) and in *batman^l(2)k02512^*/+ (24.4, ΔON = 19.0%, p<0.001) heterozygotes, when compared to Canton-S (20.5, Figure 1B Table S1B,B’). When one copy of the wild-type *batman*+ transgene (*p10*, [47]) was added back to *batman^l(2)k02512^*/+ heterozygotes, the number of ovarioles was significantly reduced (21.7, ΔON = 5.9%) when compared to that of *batman^l(2)k02512^*/+ (p<0.001), thus reverting towards a wild-type number of ovarioles (Figure 1B and Table 1SB,B’). In heteroallelic *batman* escapers (*batman^l(2)k02512^*/*batman^l(2)k07907^*) the average number of ovarioles (26.8, ΔON = 30.7%) was significantly higher than that in heterozygotes from the same experimental series (p<0.001, Figure 1B and Table S1B,B’). In addition, the number of ovarioles in *p10, batman^l(2)k02512^*/*batman^l(2)k07907^* ovaries (25.5, ΔON = 23.4%) was similar to that observed in *batman^l(2)k02512^*/+ heterozygotes (p = 0.17, Figure 1B and Table S1B,B’), indicating that the *p10* transgene rescues the weak *batman^l(2)k07907^* allele. Since the increase in the number of ovarioles is not allele-specific and since an extra copy of the wild-type *batman* gene rescues a *batman* mutant allele, we concluded that *batman* function is required for limiting the number of ovarioles. In *batman^l(2)k02512/^psq^0115^* double heterozygotes, ovariole number was significantly higher (27.6) than that observed in *batman^l(2)k02512/^+* (22.3, p<0.001) and in *psq^0115^/+* (23.5, p<0.001) heterozygotes (Figure 1C and Table S1C). In addition, 30% of the *batman^l(2)k02512^/psq^0115^* ovaries had 29 ovarioles or more, with a maximum of 32, whereas none of the single heterozygotes had more than 28 ovarioles per ovary, thereby suggesting that *batman* interacts genetically with *psq* in the negative control of ovariole number.

*Trl^81.1^* is a null lethal allele [60]. *Trl^81.1^*/+ heterozygotes had an average of 22.5 ovarioles per ovary, which corresponds to 19% (ΔON) more ovarioles than wild-type flies in the same experimental series (p<0.001, Figure 1D and Table S1D,D’). In *psq^0115^/+; Trl^81.1^/+* double heterozygotes, the number of ovarioles per ovary (24.9, ΔON = 31.7%) was higher than that of each of the single heterozygotes, *Trl^81.1^*/+ (ΔON = 19%, p<0.001) and *psq^0115^/+* (ΔON = 11.1%, p<0.001) (Figure 1D and Table S1D,D’). In addition, a new class of 29 ovarioles per ovary was observed in *psq^0115^/+; Trl^81.1^/+* female flies. This phenotypic class, which represents 20% of the ovaries of the double heterozygotes, was not observed in single *psq^0115/^+* heterozygotes, in *Trl^81.1/^+* heterozygotes or in Canton-S ovaries. A second *Trl* allele, the *Trl^13c^* hypomorphic allele [46], was also tested. *psq^0115^/+; Trl^13c^/+* double heterozygous females had 25.3 ovarioles per ovary, which is significantly higher than what was observed for single *psq^0115^/+* (23.5, p<0.01) and *Trl^13c^/+* (21.1, p<0.001) heterozygotes (Figure 1C and Table S1C). Taken together, these results suggest that *Trl* and *psq* genetically interact in the negative control of ovariole number.

When the dose of *batman* was reduced by half in *Trl^81.1^*/+ heterozygotes, the number of ovarioles was not significantly modified when compared to the *Trl^81.1^*/+ context (21.7 and 22.5, respectively, p>0.1) (Figure 1D, Table S1D). Similar results were obtained when comparing *psq^0115^/+; Trl^81.1^/+* double heterozygotes in a *batman*+ context or in a *batman* heterozygous context since the difference in the number of ovarioles observed in *batman^ l(2)k02512^ psq^0115^/+; Trl^81.1^/+* triple heterozygotes (24.2) when compared to that in *psq^0115^/+; Trl^81.1^/+* double heterozygotes (24.9) is not significant (p>0.1). Therefore, once the dose of *Trl* is reduced by half, a reduction in *batman* does not further modify the number of ovarioles.

In conclusion, our genetic analysis of the ovarian phenotype of *psq*, *Trl* and *batman* demonstrates that the three ttk-BTB group genes negatively control the number of ovarioles, and also suggest that in this process, *psq* cooperates with *Trl* and *batman*.

### Function of the *bab* Locus and Genetic Interactions with *psq*, *Trl* and *batman* in the Control of Ovariole Number

Both the presence of BTB domains of the ttk group in *bab* gene products and the requirement of the *bab* locus for TF formation and morphogenesis of the ovary [6], [7], [39] prompted us to address the interactions between the *bab* locus and *psq*, *Trl* and *batman*. Three *bab* alleles, *bab^P^*, *bab^E1^,* and *bab^PR72^* affecting differentially the levels of both BAB1 and BAB2 proteins [39], [61], as well as *bab^AR07^*, a deficiency inactivating both *bab1* and *bab2* [39] were assayed.

**Figure 3:**
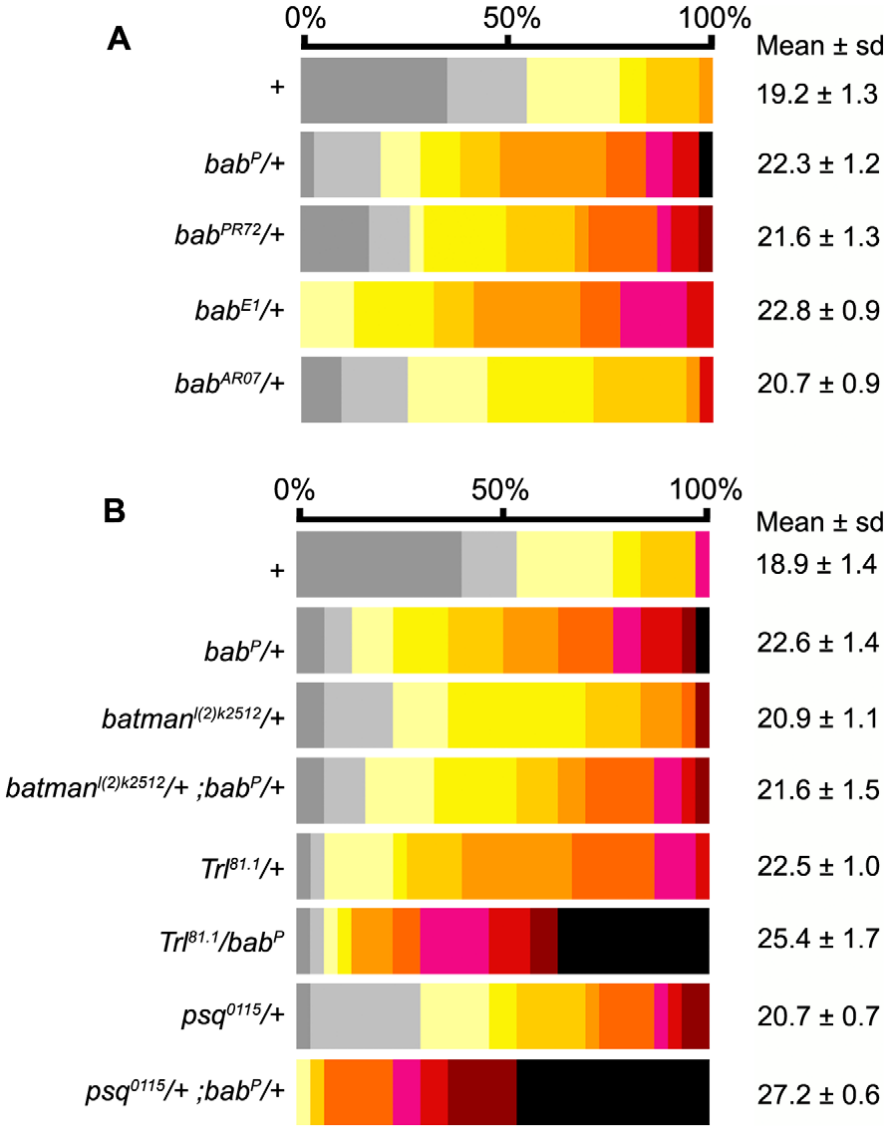
Ovariole number in *bab* mutant heterozygous combinations. (A) Distribution of the number of ovarioles per ovary in different *bab* heterozygotes. For statistics (comparison between *bab* heterozygotes and Canton-S (+)) see Table S3A. (B) Distribution of the number of ovarioles per ovary in genetic interaction assays between the *bab* locus and *psq*, *Trl* and *batman*. For statistics (comparison between different allelic combinations) see Table S3B. The mean number of ovarioles per ovary is given on the right. All values are expressed as the mean +/− sample standard deviation. Color table as in Figure 1.

As shown by Couderc et al. [39], we previously observed that ovaries from *bab^P^* and *bab^AR07^* homozygotes were strongly reduced in size and contained very few ovarioles [61]. Thus we expected that partial reduction of *bab* locus function in *bab* heterozygotes would either reduce or have no effect on ovariole number. Strikingly, we found that ovaries from *bab^P^*/+ (22.3, p<0.001), *bab^PR72/^*+ (21.6, p<0.001), *bab^E1^*/+ (22.8, p<0.001) and *bab^AR07/^*+ (20.7, p<0.01) contained significantly more ovarioles than Canton-S flies from the same experimental series (19.2, Figure 3A and Table S3A), with a relative increase (ΔON) of 16.1%, 12.5%, 18.8% and 7.8% in ovariole number, respectively, when compared to Canton-S (Table S3A’). These results suggest that like *psq*, *Trl* and *batman*, *bab* function is required to negatively control ovariole number. Double heterozygous combinations of *bab^P^* with *psq^0115^*, *Trl^81.1^* and *batman^l(2)k02512^* mutant alleles were thus generated in order to test genetic interactions between these genes (Figure 3B). Among these combinations, *Trl^81.1/^bab^P^* and *psq^0115^*/+; *bab^P^*/+ double heterozygotes had significantly more ovarioles (25.4 and 27.2, respectively) than each of the single heterozygotes (Figure 3B and Table S3B). In addition, more than one third of *psq^0115^*/+; *bab^P^/*+ ovaries had 29 ovarioles or more, whereas only one case was observed in *bab^P^* heterozygotes, and none in *psq^0115^* heterozygotes. Such interactions were not observed between *bab^P^* and *batman* since the number of ovarioles in double heterozygotes could not be distinguished from that in single heterozygotes (p>0.1, Figure 3B and Table S3B). Therefore, we conclude that *psq*, *Trl* and *bab* genetically interact to control ovariole number.

### The Number of TF cells Per Ovary and the Sorting of TF cells into TFs are Controlled Differentially by *psq* and *bab*

**Figure 4:**
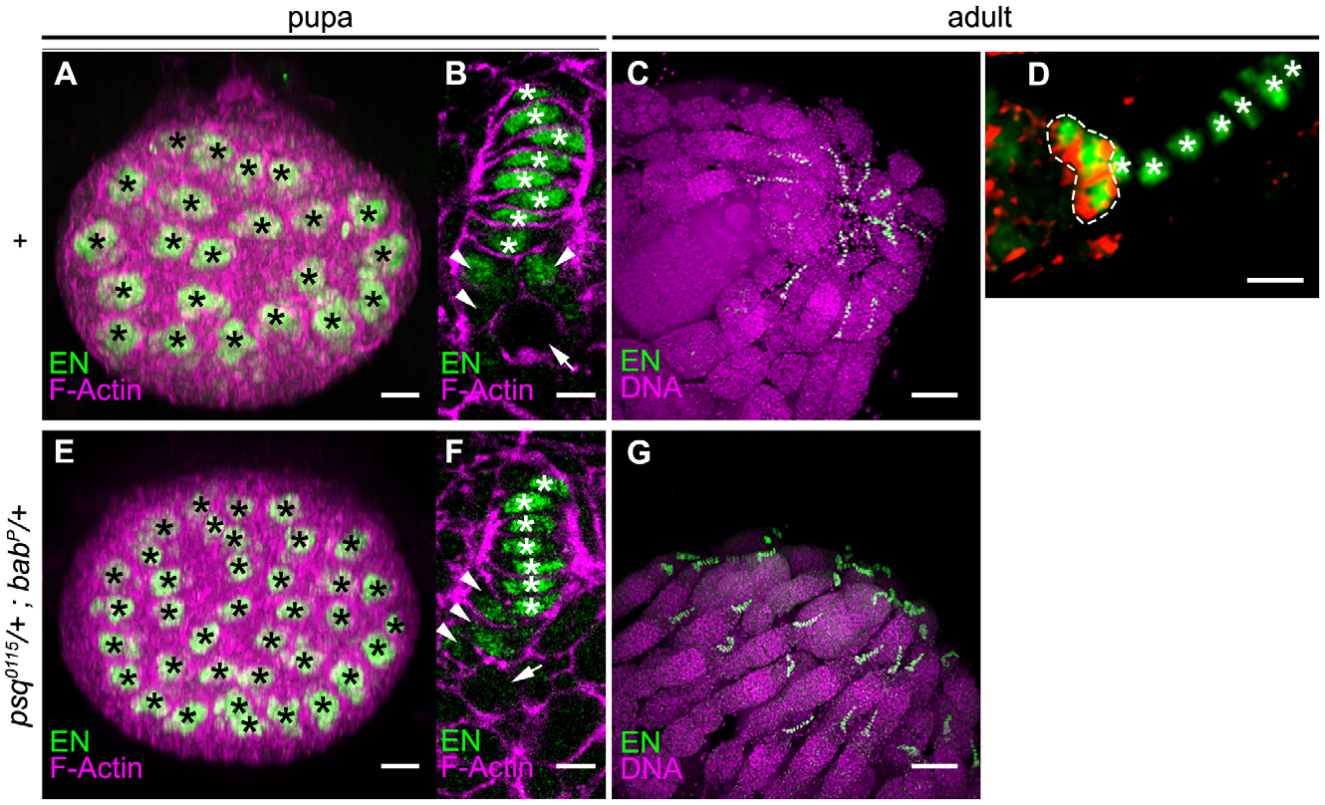
Detection of TFCs in the pupal and in the adult ovary. (A–D) Wild type control (Canton-S). (E–G) *psq^0115/^+*; *bab^P/^*+ heterozygotes. EN positive (green) cells include two cell populations, TFCs (white stars in B, D, F), and CC (arrowheads in B, F, circled by a dotted white line in D). At the early pupal stage (A, B, E, F), confocal projections through the anterior/posterior axis of ovaries (A, E) allows counting of the number of TF per ovary (each TF is marked by a black star). Sections through individual early pupal TFs (B, F) allow discrimination between TFCs with a flattened nucleus (white stars) and CCs with a more rounded nucleus (arrowheads in B, F) located posterior to TFCs. Germ cells are marked by an arrow. In one-day-old flies (C, D, G), counting of TFs and TFCs was performed on confocal 3D stacks from whole mount ovaries stained with anti-En (green), anti DE-Cadherin (red, in D) and TO-PRO-3® (purple, in D, G). TFs protrude at the tip of the germarium. In the germarium, CCs (circled by a dotted white line) accumulate high levels of DE-cadherin (red), whereas the adjacent basal TFCs do not. Anterior to the right. Scale bar : (A, E) 10 µm, (B,F) 2.5 µm, (C, G) 40 µm, (D) 5 µm.

We next addressed the role of ttk-BTB group factors in the control of cell number and cell sorting into TFs. We focused on *psq* and *bab* mutants and determined cell number in individual TFs and in the whole TFC population, as well as the size of individual TFCs (Figure 4 and Materials and Methods). In early pupae (1–2 hours after puparium formation), TF formation is completed in the wild-type [1], [2]. The following parameters were thus measured at the early pupal stage: i) the number of TFCs per TF (TFC/TF), ii) the number of TFCs per ovary (TFC/O), and iii) the mean volume of TFCs (TFCV).

**Figure 5:**
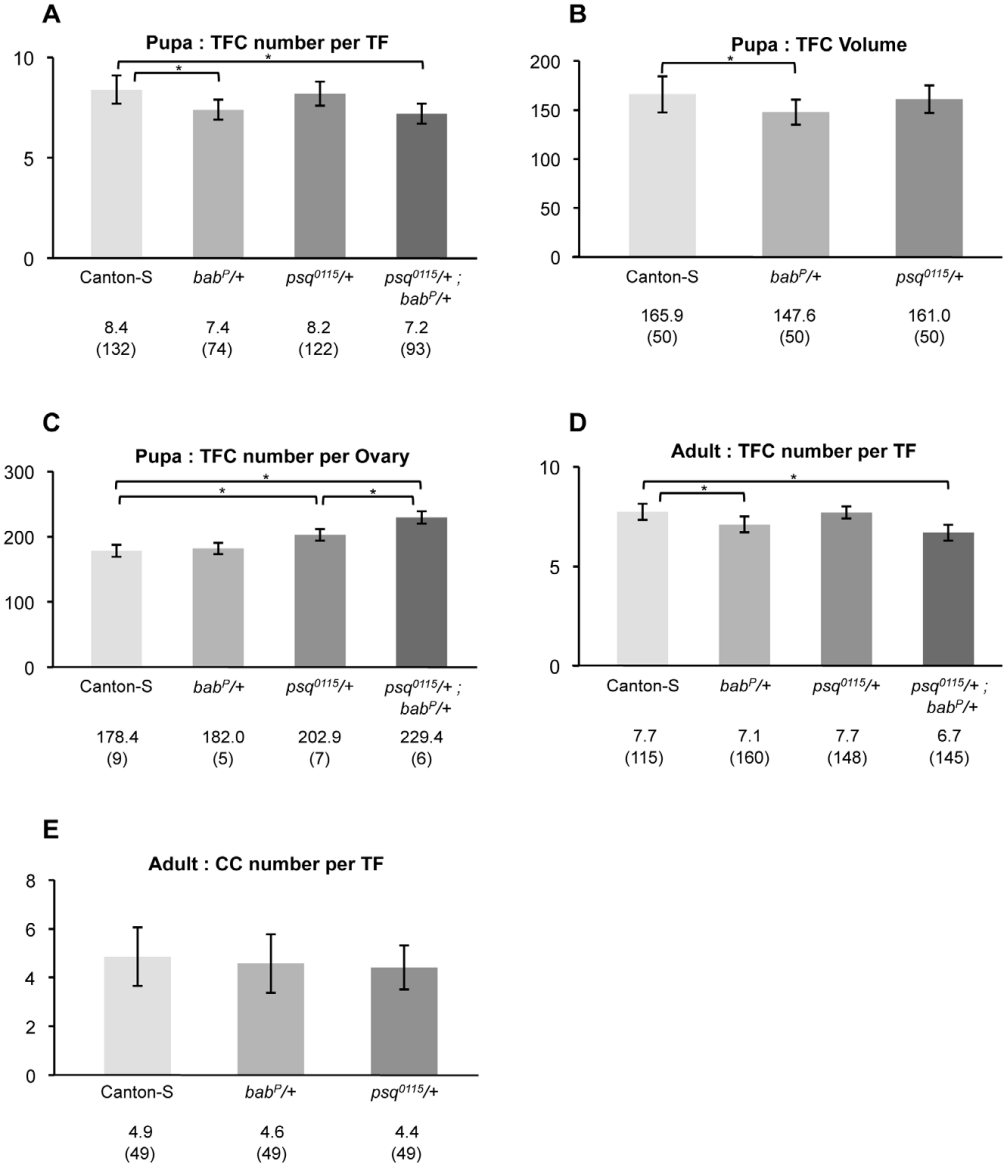
Quantitative analysis of cell number and size in *psq* and *bab* heterozygotes. (A) TFC number per TF at the early pupal stage. (B) TFC volume at the early pupal stage. (C) TFC number per ovary at the early pupal stage. (D) TFC number per TF in adults. (E) CC number per germarium in adults. Canton-S flies were used as a wild-type reference. Genotypes are given below each chart. In heterozygotes, +indicates wild type chromosomes from the Canton-S stock. For each genotype, values are expressed as the mean number (A, C–E), or mean volume (B) and sample size is given in parenthesis. Significant differences between genotypes are marked by a bracket and asterisk. All p values are given in Table S4.

In *psq^0115^*/+ heterozygotes, TFC/TF (Figure 5A) and TFC volume (Figure 5B) were similar to those observed in the wild type (p>0.1, Table S4A and S4B), while TFC/O (Figure 5C) was higher (202.9) than that in the wild type (178.4, p<1.9 10^−2^, Table S4C). These results indicate that in the developing pupal ovary, *psq* negatively controls the total TFC number per ovary, thereby suggesting that *psq* regulates directly or indirectly the proliferation and/or specification of TFCs. The increase of 24.5 TFCs per ovary in *psq^0115^*/+ heterozygotes is sufficient to account for the 3.4 extra TFs (Table S5) each containing 8.2 TFC/TF, a number that is not different from the 8.4 TFC/TF found in the wild type (Figure 5A, Table S4A).

In *bab^P^*/+ heterozygotes, TFC/O (Figure 5C) was similar to that in the wild type (p>0.1, Table S4C). In contrast, both TFC/TF (7.4, p<0.001, Figure 5A) and TFC volume (147.6, p<0.01, Figure 5B) were lower than those observed in the wild type (Table S4A and Table S4B). Thus when *bab* function is reduced, compared to the wild type context, fewer TFCs appear to pile up in order to form individual TFs and consequently, more TFs are formed. Indeed, in the *bab^P^*/+ heterozygotes, there was on average one less cell per TF than in the wild-type, which given the mean 24.6 ovarioles per ovary in *bab^P^*/+ females, can account for the 3.4 extra TFs with a mean 7.4 TFC/TF. Unexpectedly perhaps, the reduction in cell number per TF in *bab^P^*/+ heterozygotes was not correlated to an increase in TFC volume. In fact, TFC volume was lower in *bab^P^*/+ TFs than in the wild type (p<0.01, Table S4B). Therefore, it appears that *bab* function regulates TF morphogenesis through integration of a minimum number of cells per TF, and this regulation does not appear to “sense” the final size (volume) of individual TFs.

In *psq^0115^*/+; *bab^P^*/+ the number of TFCs per TF (7.2, Figure 5A) was similar to that observed in the *bab^P^*/+ context (7.4, p = 8.1 10^−2^, Table S4A). This result suggests that *psq* has little or no contribution to the process that controls the number of TFCs per TF, even when *bab* levels are reduced. In contrast, the number of TFCs per ovary in *psq^0115^*/+; *bab^P^*/+ (229.4, Figure 5C) was higher than that in *psq^0115^*/+ single heterozygotes (202.9, p = 2.5 10^−2^, Table S4C). This result is not to be expected if *bab* and *psq* functions are independent (p<0.01). The increase of 26.5 TFCs per ovary in *psq^0115^*/+; *bab^P^*/+ double heterozygotes compared to *psq^0115^*/+ only accounts for an expected 3.7 extra TFs, whereas we observe that *psq^0115^*/+; *bab^P^*/+ ovaries contain a mean 7.2 extra TFs per ovary (Table S5A,B) each containing 7.2 TFC/TF, a number that is not different from the 7.4 TFC/TF found in *psq^0115^*/+ simple heterozygotes (Figure 5A Table S4A). Since reducing the dose of *bab* enhanced the increase in the number of TFCs found in *psq* heterozygotes, we conclude that in addition to its requirement for the definition of the correct number of TFCs per TF, *bab* function, in interaction with *psq,* is also required for controlling the total number of TFCs.

We further investigated the increase in ovariole number by measuring the number of TFCs per ovariole in adults of the Canton-S, *psq^0115^*/+, *bab^P^*/+ and *psq^0115^*/+; *bab^P^*/+ genotypes (Figure 5D). In *bab^P^*/+ and *psq^0115^*/+; *bab^P^*/+ adult females, there is less TFC per TF (7.1 and 6.7 respectively) than in the Canton-S control (7.7, p<0.001) (Figure 5D and Table S4D). In contrast, TFC/TF in *psq^0115^*/+ adult females (7.7) is similar to that in the control (Figure 5D and Table S4D). Thus, in adults, the variation in TFC/TF depending on the genotype is similar to that observed at the pupal stage. This result indicates that the lower TFC/TF observed at the pupal stage for *bab^P^*/+ and *psq^0115^*/+; *bab^P^*/+ ovaries compared to the control is not due to a developmental delay that is compensated for by the adult stage.

In order to address the possibility of a general function of *psq* and *bab* in the control of cell number, we compared the number of cells of a second cell type, *i.e.* CCs, between Canton-S, *psq^0115^*/+ and *bab^P^*/+ simple heterozygotes. Like TFCs, CC express Engrailed in pupae and adults, but can be distinguished from TFCs in adults since they express higher levels of DE-Cadherin ([62], Figure 4D). We found that the number of CCs per TF (Figure 5E) was similar in the wild type, *psq^0115^*/+ and *bab^P^*/+ context (p>0.1, Table S4E). This result indicates that, within the germline stem cell niche, the effect of both contexts are specific to TFCs and do not extend to CCs.

In order to address a possible effect of *bab* and *psq* mutations on cell proliferation, the number of cells undergoing mitosis was compared in whole mount L3 ovaries of Canton-S and *psq^0115^*/+; *bab^P^*/+ double heterozygotes using Ser10 PhosphoH3 antibodies as a marker for chromosome condensation (Figure S2). For both genotypes, mitotic figures in somatic cells were found in two broad anterior and posterior domains, excluding the equatorial EN-positive TFCs. No difference was detected at this stage in the mean number of mitotic figures per ovary between the two genotypes (56.1 for Canton-S and 56.5 for the double heterozygote, p = 0.94 and n = 10). This result suggests that during the morphogenetic wave, the early phase of ovarian morphogenesis at L3 during which TFCs are specified and sort out, the dynamics of mitosis are equivalent in Canton-S and *psq^0115^*/+; *bab^P^*/+ double heterozygotes. In contrast, the number of TF that form as well as the number of TFCs per ovary is greater in the *psq^0115^*/+; *bab^P^*/+ double heterozygotes. This result is compatible with a function for these genes in limiting TFC specification and/or in the proliferation of TFC precursors.

## Discussion

In a compound organ, the control of final size relies on the control of the number of units produced, as well as on the size of each unit. The *Drosophila* ovary provides a good system to identify genetic pathways and cellular mechanisms that, during development, control the number of units (ovarioles) composing the adult organ (ovary). Here we show that in *D. melanogaster* this number is under the control of several nuclear BTB factors of the ttk group [44] that share high sequence similarity and the capacity to form heterodimers [44], [47], [49]. It is noteworthy that three of the corresponding genes, i.e. *psq* (47A13–47B1), *Trl* (70F4-70F4) and *batman* (55B9-55B9), map in QTL that were identified in inter-specific studies as controlling ovariole number [31]. In contrast, they do not map in intervals defined for QTL involved in intra-specific variation [33] or in QTL involved in phenotypic plasticity in *D. melanogaster* [30]. Our study focused on the determination of TF number in early pupa, which prefigures the number of ovarioles that will be produced [1], [2].

Interestingly, *bab*, *batman*, and *Trl*, as well as other chromatin regulators, were previously shown to participate to a genetic network that controls the effect of temperature on abdominal pigmentation in *D. melanogaster* [63]. Together with the work presented here, these results suggest the possibility that a regulatory network constituted of BAB and chromatin regulators (PSQ, Batman, and TRL/GAF) may have a broad impact on the regulation of several quantitative traits and phenotypic plasticity in *D. melanogaster.* However, despite the common biochemical properties of PSQ and BAB ttk group BTB factors, we found that during development in the ovary, these factors do not play identical functions since *psq* controls the total number of TF cells per ovary, whereas *bab* is mainly involved in controlling the number of TF cells in individual TFs.

### Complexity of the BTB Network Controlling Ovariole Number

The three genes *Trl* [46], *psq* [51], and *batman* [47], [50], [64] were previously characterized in the regulation of Hox genes. In this context, a simple model for their molecular function is that PSQ-A, a PSQ isoform that contains a BTB domain, and TRL/GAF, also containing a BTB domain, bind GA repeats present in the PREs through a similar DNA-binding domain [65]. PRE binding of TRL/GAF and PSQ may lead to the recruitment or stabilization of other Polycomb or Trithorax group factors that participate to the epigenetic transcriptional memory of the homeotic loci (reviewed in [66]). Batman, a small protein which only contains a BTB domain, does not bind DNA. However it binds to TRL/GAF through its BTB domain and thereby also associates to the GA repeats in the PREs together with the PRC1 protein PH [47], [50]. In addition, Batman and PSQ BTB domains interact in the yeast 2-hybrid system [44]. It is thus possible that GA-binding complexes contain various combinations of the three BTB factors PSQ-A, TRL/GAF and Batman. The functional significance of the different combinations remains to be addressed.

In the ovary, we found that the three genes, *psq*, *Trl* and *batman* negatively control the number of ovarioles. In addition, we showed that *psq* interacts genetically with both *Trl* and *batman* in the control of ovariole number. This result is consistent with the hypothesis of cooperation between PSQ, TRL/GAF and Batman proteins for formation of complexes regulating target genes. However, genetic interactions could not be detected between *batman* and *Trl* for the control of ovariole number. Therefore, the nature of the TRL/GAF-Batman complexes may be such that the effect of removing one dose of either one of these components cannot be aggravated by removing a dose of the second component.

The *bab* locus is also involved in limiting ovariole number, suggesting that it plays at least two functions in ovarian morphogenesis. The first one, previously described, is for the correct specification of TFCs and the formation of TFs, as evidenced by the fact that when *bab* gene products are absent or strongly reduced, only few TFCs are present, TFs cannot form and the ovary remains rudimentary [6]. A second role, shown here, is for negative control of ovariole number by determining the number of TFCs sorting out and forming TFs. Therefore, in the absence of function of the *bab* locus, TFCs do not seem to be specified correctly, and the few TFCs that are present do not sort out correctly [6]. When one dose of the *bab* locus is present, there seems to be sufficient levels of *bab* proteins to specify TFCs, however not sufficient to allow enough TFCs to sort out and form individual TFs, leading to formation of a greater number of shorter TFs. It is noteworthy that the *bab* locus contains two paralogues, *bab1* and *bab2*, expressed in two different patterns in the larval ovary. Indeed *bab2* is expressed in all somatic cells, with a stronger expression in TFCs, whereas *bab1* expression is limited to TFCs [39]. Further studies will be necessary to address whether during evolution, separate functions were attributed to the two paralogues *bab1* and *bab2* in morphogenesis of the ovary.

Genetic interactions between *bab* and *Trl* and between *bab* and *psq* have a major effect on ovariole number, since in double heterozygotes, up to 34 ovarioles per ovary may be formed, which is never observed in the wild type or in single heterozygotes. These interactions are restricted to the negative regulation of ovariole number in the ovary, since, unlike for *bab*, none of the *Trl*, *psq* or *batman* mutations lead to rudimentary or reduced ovaries, even in double heterozygous combinations with a deficiency of the *bab* locus. Interactions between the *bab* locus and *psq* or *Trl* in limiting the number of ovarioles may be the result of various molecular processes. The fact that they belong to the ttk-BTB group supports the hypothesis of their participation to common complexes [44] acting on common target genes, but this issue remains to be addressed *in vivo*. Alternatively, but not exclusively, regulatory interactions may exist between these genes at the transcriptional level. Indeed, a Polycomb Responsive Element (PRE) was recently detected in the sequence of the *bab* locus [67]. However, when analyzed at the cellular level, *psq* and *bab* phenotypes are different since they act on TFC number and TFC sorting out, respectively. Therefore, the increase in ovariole number observed when the dose of *psq*, *batman* or *Trl* is reduced, as well as the genetic interactions between *psq* and *bab* are unlikely to be the consequence of regulation of the *bab* locus by the Polycomb/Trithorax Group factors PSQ, Batman and TRL/GAF. Since *batman* and *Trl* were found previously to interact with the *bab* locus in the control of the ectopic sex comb phenotype of *bab^AR07^* heterozygotes [52], our results suggest that interactions between the *bab* locus and other ttk group BTB genes may have a broad impact on several quantitative traits in *Drosophila*. However, the specificity of the phenotypes (*i.e.* presence of rudimentary ovary only in *bab* mutants, cellular processes affected differentially by *bab* and *psq* in TF formation) underscores the functional complexity of the network of ttk group BTB factors that may also depend on the cell type.

### Genetic Control of Ovariole Number in *Drosophila melanogaster*

The morphogenetic wave that leads to TF formation occurs after a last mitotic wave among TFC precursors in the third instar larval ovary. Morphogenesis of TFs is thus considered as a post-mitotic process [8]. The total number of ovarioles in *D. melanogaster* correlates with that of TFs in early pupae, suggesting that at the pupal stage, the process that defines ovariole number is completed [2]. The number of units in the compound ovary may thus be determined by the initial cell number in the ovary at the onset of morphogenesis [2] and possibly by cell death [31], as well as by the efficiency of recruiting new units during the morphogenetic wave, before pupariation. The elimination of supernumerary units when too many have been made, as is the case in honeybees [68] does not appear to be a major mechanism controlling ovariole number in *D. melanogaster* [2]. Anatomical studies in the *D. melanogaster* subgroup show that ovariole number varies widely between species and shows phenotypic plasticity in *D. melanogaster* [2], [23], [24], [25]. Since *psq*, *Trl*, and *batman* map cytogenetically in the same location as QTL that were identified as controlling the difference of ovariole number between two different species of the *melanogaster* subgroup [31], they constitute possible candidates that may be involved in the evolution of ovariole number in this group. Whereas inter-specific as well as diet-dependent intra-specific variation correlate with differences in cell number in the ovary primordium, thermal phenotypic plasticity appears to be a post-mitotic mechanism that operates on the dynamics of the morphogenetic wave [2] and cell-cell sorting [34] at the late third instar in larvae. These dynamics are under the control of ecdysone since in *Df(EcR)* heterozygotes, TF formation is delayed, and the number of ovarioles is reduced [69]. Gancz et al. [36] showed that indeed, *broad* under the control of EcR/USP determines a temporal window during which TF morphogenesis occurs.

At the cellular level, the presence of more TFs in the adult ovary may, in theory, result from at least three different mechanisms [34]:

1. cell sorting: starting with a fixed number of TFC precursors, more TFs may form when fewer TFCs sort and pile up to form each TF;
2. cell proliferation and/or specification of TFC: starting with an amplified population of TFC precursors, if the number of TFCs per TF is constant, a higher number of TFs may form;
3. control of the size of individual TFs: starting with a fixed number of TFC precursors, if there is a constraint on the size/volume of individual TFs, increasing cell size would lead to the formation of more TFs each containing less TFCs.

Our study allows us to propose a genetic basis supporting two of these mechanisms, cell sorting under the control of the *bab* locus, and control of TFC number under the negative control of *psq*. Two alternate hypotheses may explain a higher number of TFC in ovaries from *psq* heterozygotes: 1) increased proliferation of TFC precursors, and 2) increased specification of TFC precursors from “naïve” somatic cells. These two hypotheses could not be distinguished in our experiments. In fact, we found that *ca*. 55 cells are in a proliferative state in mid- to late-third instar wild type ovaries, and that this proliferation is not affected either regionally or globally in double heterozygotes for *psq* and *bab* in which the mean TFC number per ovary is the highest. However, the difference in the mean number of TFCs between wild type and *psq* heterozygotes is 27 cells. Considering that TFC specification occurs over a 24 hour period, about one supplementary cell entering in mitosis per hour would be sufficient to explain the increase in the number of TFCs, which would probably not be detectable in our assay. Therefore, the mechanism underlying control of TFC number by *psq* in the ovary remains undetermined. If *psq* functions in limiting proliferation in the ovary, this would be the opposite of what has been described previously in the retina, where *psq* overexpression enhances proliferation [59]. Considering the fact that PSQ binds several hundred sites on polytene chromosomes [49], it is likely that the resulting effect of its up- or down-regulation comes from the combination of factors that are transcriptionnally controlled by this epigenetic regulator, a combination that may vary depending on the cell-type and context.

We also found that in *psq* as well as in *bab* heterozygotes, the number of CCs per ovariole was not affected whereas TF number increased, suggesting that the number of CC per ovary globally increases with that of TFs, independently of the mechanisms that lead to the increase in TF number. Whereas our study did not directly address this issue, these results suggest that downstream of TF formation, a developmental program controls the integration of a constant number of cap cells per TF, independently of the number of TFCs per TF and independently of the total number of TFs, which is in agreement with the hypothesis that TFs are organizing centers for each ovariole as it forms. Indeed, a current model proposes that the basal TFC induces adjacent somatic cells located posteriorly to engage into the CC fate [14], under the control of the Notch/Delta signalization [13], [14]. When more TFs are made, each TF may function as a unit in which the basal TFC may thus play a local role in inducing CCs, independently of the total number of TF in the whole ovary.

It was shown that TF number is controlled differentially at the inter- or intra-specific levels [34] by at least two processes. Variation of TFC number correlates with intra-specific, diet-dependent variation of TF number as well as with inter-specific differences inside the *melanogaster* sub-group. In contrast, control of the sorting of TFCs into TFs is involved in the intra-specific temperature-dependent variation in ovariole number [34]. Paralleling these observations, we found that nuclear BTB factors of the ttk group regulate ovariole number at these same two levels, but at least partially separately: *psq* negatively controls the total number of TFCs per ovary, whereas *bab* ensures that a minimum number of TFCs are incorporated per TF, thereby affecting the sorting of TF precursors. One hypothesis is that *psq* may be involved in intra-specific diet-dependent variation and inter-specific variation of ovariole number. It will be also interesting to address whether *psq* controls the Hippo and/or mTOR/S6K pathways since they have also been shown to affect ovariole number by increasing TFC number [34]. The *bab* locus alone does not appear to control TFC number per ovary, however in interaction with *psq* it appears to also modify TFC number, in addition to its role on the sorting of TFCs into TFs. Gibert et al. [52] showed that *bab* in interaction with epigenetic regulators including *batman* controls temperature-dependent plasticity of abdominal pigmentation. The *bab* locus is also known to play a key role in inter-specific variation of sexually dimorphic abdominal pigmentation [70], [71], as well as in thermal phenotypic plasticity in association with other chromatin regulators (*cramped*, *corto*) [52], [72]. Future work should help determine the role of different ttk-BTB group factors in environmental plasticity and in inter-species variation.

## Materials and Methods

### Fly Stocks

Flies were grown on standard corn-agar medium at 25°C. Mutant alleles were kind gifts from J-L. Couderc (*bab2^E1^*, *bab-Gal4^B8^* referred to here as *bab-Gal4*), M. Boube (*bab^AR07^*), D. Godt (*bab^P^*), C.Berg (*psq^0115^* and *psq* ^Δ*18*^), and A. Greenberg (*Trl^81.1^*). The *p10* transgene is a *batman+* genomic construct that rescues *batman^l(2)k02512^* lethality [47]. *Trl^81.1^* is a null *Trl* allele [60]. *bab^P^* is a *P*-Element insertion in the *bab1* gene. *bab^PR72^* and *bab^AR07^* are *P*-Element imprecise excisions from *bab^P^* and *bab^A128^*, respectively. *bab^E1^* is an EMS mutant affecting both *bab1* and *bab2* [39], [61]. The original *bab^AR07^* allele was previously outcrossed to remove a second lethal mutation on the same chromosome (Muriel Boubé, personal communication). The two *bab* alleles *babP* [6] and *bab^AR07^* (F. Laski, unpublished work [70]) were generated in independent mutagenesis, and thus have different genetic backgrounds. The dsRNA line *UAS-psqIR* (2368R-1) was provided by the National Institute of Genetics Fly Stock Center. This construct has potential off-targets (OTs, as defined by the presence of 16-mers that are shared between OTs and *psqIR* sequences [73]) but despite the fact that it covers the BTB-encoding sequence from *psq*, it does not target the *lola* locus, adjacent to *psq* and encoding several BTB-containing isoforms. The *UAS-psqIR* thus allows knockdown of *psq* function without affecting the expression of the neighboring locus *lola* affected in the *psq* ^Δ*18*^ allele.

### Phenotypic Analysis of Pupal and Adult Ovaries and Statistics

Balanced stocks were outcrossed to the wild type Canton-S stock to eliminate possible effects of the balancers and heterozygous female flies were scored for the number of ovarioles, and compared to Canton-S flies from the same experimental series. Outcrossing to the Canton-S wild type strain reduces the influence of genetic background by exchanging half of the original chromosomes with Canton-S chromosomes from the control stock. Double heterozygotes were compared to each of the single heterozygotes. Crosses were performed under uncrowded conditions at 25°C. In all cases where single heterozygotes were generated, females were from the Canton-S wild type. Five females were crossed to the males of the appropriate genotype and allowed to lay eggs for a 12 hours period on medium with live yeast, after which they were flipped to a fresh tube. In different experimental series, Canton-S females had a mean number of ovarioles per ovary that ranged from 19 to 20.5, likely depending on fluctuations of growing conditions. Since ovariole number depends upon environmental cues, each experimental series included its appropriate control in a parallel cross.

For all experiments in which the distribution of ovariole number per ovary was quantified (Figures 1, 2, 3), the entire progeny was harvested to avoid bias due to slower development of individual with different genotypes. In preliminary experiments, left and right ovaries from wild type Canton-S flies were compared in order to address a possible correlation in the number of ovarioles between the two ovaries of the same individual. We found no linear correlation between the number of ovarioles between ovaries of the same pair (n = 105, p<0.01). Therefore, we pooled all dissected ovaries from all individuals for each experimental point. At least 20 females aged from one to three days were sampled and 30 randomly-picked ovaries were analyzed for each point of a series, except in the series where *psq* escapers were assayed (20 ovaries analyzed). Ovaries were fixed in PBS, 3.7% formaldehyde during 30 minutes at room temperature and stored at 4°C before observation under a stereoscopic microscope (Leica MZFL III). DAPI-stained (5 µg/ml in PBS) ovaries were then transferred individually into a drop of PBS/Glycerol (1/1) on a glass slide and ovarioles were separated in order to count germaria under epifluorescence (Leica DMRD). The number of germaria was scored to quantify the number of ovarioles per ovary, irrespective of possible defects in oogenesis. The relative variation of ovariole number (ΔON) was calculated as the ratio of the difference in ovariole number between the sample and Canton-S to the number of ovarioles in Canton-S in the same experimental series. Statistical analysis was performed using a Student’s t-test to compare the mean number of ovarioles between different genotypes. All p values are given in Table S1, S2, S3, S4, S5.

In the *psq* knockdown experiment using RNAi, Canton-S was used as a wild-type control. The *bab-Gal4* line and the *UAS-psqIR* RNAi line were crossed to Canton-S and the female progeny was scored for the mean number of ovarioles per ovary and compared to the wild-type control from the same series using a Student’s t-test, showing no significant difference (Figure 2 and Table S2). In contrast, *UAS-psqIR; bab-Gal4/+*had significantly more ovarioles per ovary than Canton-S. 2-way ANOVA was performed on the set of data under a multiplicative model (p values are given in Table S2), allowing us to conclude that the higher number of ovarioles per ovary in *UAS-psqIR; bab-Gal4/+* flies did not result from a background effect of the driver and the UAS-RNAi line but rather from the induction of the UAS/Gal4 system.

For the quantification of the number of TF cells per TF in adults, due to the fact that TFs are fragile structures that may be broken-off and lost during dissection, counting was performed on 3D confocal stacks from whole mount adult ovaries after anti-En immunofluorescence and TO-PRO-3® (Invitrogen) staining of nuclei (5 µM in PBS 0,3% Triton). Since the number of TFCs per ovariole decreases after eclosion [21], [37], all data from adults were collected from staged, one-day-old flies. A minimum of 5 ovaries were analyzed per point. The total number of TFs analyzed for each genotype is given in Table S1. All p values (Student’s t-test) are given in Table S4.

For the quantification of the number of TFs and the number of TFCs in early pupae, white pupae were selected and allowed to mature for one hour at 25°C before ovaries were dissected. Whole mount ovaries were treated with anti-En and Atto647N-phalloidin (Fluka, 100 nM) for F-Actin detection. Counting was performed on 3D confocal stacks of whole mount pupal ovaries. A minimum of 5 ovaries and a minimum of 10 TFs per ovary were analyzed for each genotype. The total number of TFs analyzed for each genotype is given in Table S5. Quantification of cell volume was performed according to Sarikaya et al. (2012) [34]. Briefly, the area of individual cells from phalloidin-stained ovaries was delimited manually and the surface occupied by a given cell in each adjacent confocal section was measured using the NIH ImageJ software. Volume refers here to the sum of all areas covered by a given cell multiplied by the step between adjacent sections (1 µm) and expressed in µm^3^.

### Immuno-fluorescence and Imaging

Whole fat bodies from female larvae or ovaries from one-day old female flies were fixed and stained using standard procedures. The following antibodies were used: rabbit anti-BAB1 (1 :1000, gift of T. Williams) [71], rat anti-BAB2 (1 :1000, gift of J.-L. Couderc) [39], rabbit anti-PSQ (1 :200; AS1, gift of M. Lehmann), rabbit anti-TRL/GAF (1 :200; gift of J. Lis), rabbit anti-Batman (1 :200) [47], mouse anti-EN (4D9, 1 :800, DSHB), mouse anti-E cadherin (DCAD2, 1 :200, DSHB). The AS1 antibody [45] allows detection of both PSQ-A and PSQ-B isoforms. Alexa 488, 568 or 647 secondary antibodies (1∶500, Molecular Probes) were incubated for 2 hours at room temperature in the presence of fluorescent phalloidin (Fluka, 100 nM). Ovaries were mounted in Citifluor (AF1, Biovalley, FR) and observed under an inverted confocal microscope NIKON TE2000-U. Laser power and gain settings were adjusted on control ovaries for each experimental series with minimum pinhole opening, and applied on test ovaries. Data were analyzed with ImageJ (NIH) and Photoshop CS2 (Adobe) softwares, using identical settings for all samples of the same experimental series.

For quantification of mitotic figures, the anti phospho-Histone H3 (Ser10) antibody (Millipore) was used (1/500) to detect mitotic cells, together with anti-En to detect TFCs. Whole mount ovaries were scanned and the total number of mitotic figures as detected with anti phosphoH3 immuno-staining was measured using the *cell counter* pluggin in ImageJ. Data were collected on 10 ovaries for each genotype.

## Supporting Information

Figure S1 **Protein levels of PSQ-A vs PSQ-B in the ovary of** ***psq^0115^/psq*** ^Δ***18***^ **female flies (0115/**Δ**18) compared to that in wild-type Canton-S (+).** Whole protein extracts corresponding to one ovary for each genotype were processed using standard western blot procedures with anti-PSQ AS1 antibody, and revealed using Supersignal West Pico Chemiluminescent (Pierce, #34084). Two major isoforms A and B (arrows) are detected in the wild type, whereas only PSQ-B is detected in *psq^0115^/psq* ^Δ*18*^ ovary. The position of molecular weight markers (Biorad) is indicated on the left of the pannel.
(TIF)

Figure S2 **Number of dividing cells in wild type Canton-S (+) and** ***psq^0115^/+; bab^P^/+*** **in ovaries from wandering larvae.** Mitotic figures were revealed using anti phospho-histone H3 (Ser10) antibody on whole mount ovaries. Confocal 3D projections was used to count mitotic cells in 10 ovaries for each phenotype.
(TIF)

Table S1 **Statistical significance (p) of comparisons between the mean number of ovarioles in heterozygous combinations of psq, Trl and batman alleles and that of wild type control flies.**
(PDF)

Table S2 **Statistical significance of comparisons between the mean number of ovarioles in bab>psqIR flies and in control flies.**
(PDF)

Table S3 **Statistical significance of comparisons between the mean number of ovarioles in combinations of bab and other ttk-BTB group mutations.**
(PDF)

Table S4 **Statistical significance for comparisons of cellular parameters between pupal or adult ovaries between heterozygous combinations of bab and psq mutations.**
(PDF)

Table S5 (PDF)
