# Supplementary figures and images for: Genetic Basis for Developmental Homeostasis of Germline Stem Cell Niche Number: A Network of Tramtrack-Group Nuclear BTB Factors
Source: PLoS One. 2012 Nov 21;7(11):e49958. doi: 10.1371/journal.pone.0049958 (PMC3503823; doi:10.1371/journal.pone.0049958)

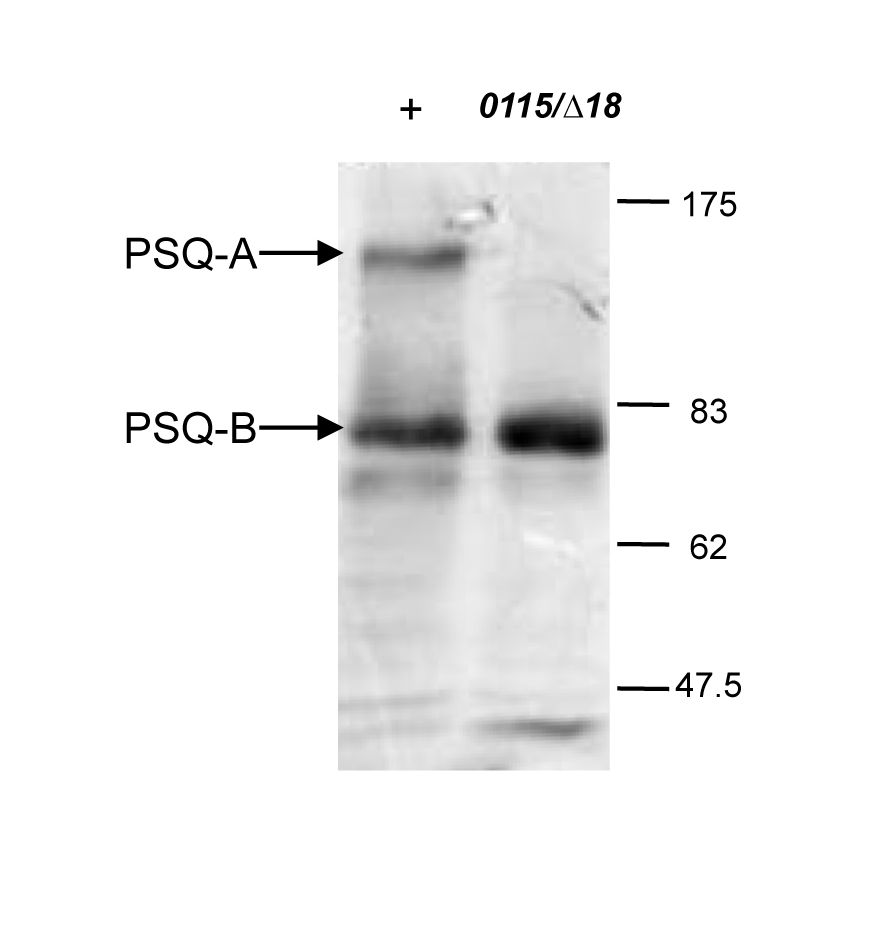

Supplement: Figure S1 — Protein levels of PSQ-A vs PSQ-B in the ovary of psq0115/psq Δ18 female flies (0115/Δ18) compared to that in wild-type Canton-S (+). Whole protein extracts corresponding to one ovary for each genotype were processed using standard western blot procedures with anti-PSQ AS1 antibody, and revealed using Supersignal West Pico Chemiluminescent (Pierce, #34084). Two major isoforms A and B (arrows) are detected in the wild type, whereas only PSQ-B is detected in psq0115/psq Δ18 ovary. The position of molecular weight markers (Biorad) is indicated on the left of the pannel. (TIF) [file pone.0049958.s001.tif]

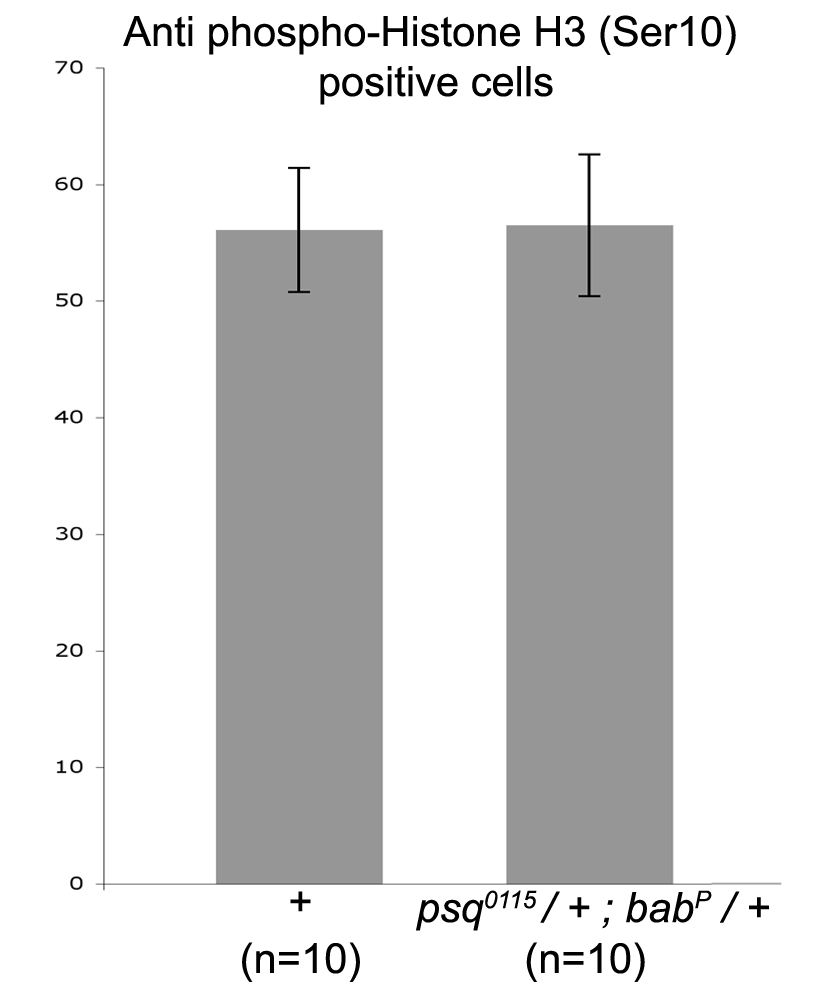

Supplement: Figure S2 — Number of dividing cells in wild type Canton-S (+) and psq0115/+; babP/+ in ovaries from wandering larvae. Mitotic figures were revealed using anti phospho-histone H3 (Ser10) antibody on whole mount ovaries. Confocal 3D projections was used to count mitotic cells in 10 ovaries for each phenotype. (TIF) [file pone.0049958.s002.tif]
