# Supplementary material for: Genetic Basis for Developmental Homeostasis of Germline Stem Cell Niche Number: A Network of Tramtrack-Group Nuclear BTB Factors
Source: PLoS One. 2012 Nov 21;7(11):e49958. doi: 10.1371/journal.pone.0049958 (PMC3503823; doi:10.1371/journal.pone.0049958)
Supplement: Table S1 — Statistical significance (p) of comparisons between the mean number of ovarioles in heterozygous combinations of psq, Trl and batman alleles and that of wild type control flies. (PDF) [file pone.0049958.s003.pdf]

Table S1 . Statistical significance (p) of comparisons between the mean number of ovarioles in heterozygous combinations of *psq, Trl* and *batman* alleles and that of wild type control flies.

For experimental series corresponding to genotypes shown in Figure 1, p-value (p) was calculated using Student's t-test between samples of appropriate genotypes (line/column in S1A-S1D). ΔON (in %, S1A', B', D') was calculated relative to Canton-S. p-values below 5% appear in red, p-values above 5% appear in green.

Table S1A : p-values (p)

	Canton-S	*psq*^{0115}/+	*psq*$^{\Delta 18}$/+
psq^{0115}/+	1,1E-09	-	-
psq$^{\Delta 18}$/+	7,1E-03	-	-
psq^{0115}/*psq*$^{\Delta 18}$	1,6E-12	5,4E-02	4,0E-07

Table S1A' : ΔON (%)

	Canton-S
psq$^{\Delta 18}$/+	7.3
psq^{0115}/+	19.0
psq^{0115}/ *psq*$^{\Delta 18}$	24.1

Table S1B : p-values (p)

	batman$^{l(2)k02512}$/+	*batman*$^{l(2)k07907}$/+	*batman*$^{l(2)k02512}$ / *batman*$^{l(2)k07907}$	*p10 batman*$^{l(2)k02512}$ / +	*p10 batman*$^{l(2)k02512}$ /*batman*$^{l(2)k07907}$
Canton-S	2.2E-10	1.1E-05	2.8E-12	8.2E-03	3.7E-10
batman$^{l(2)k02512}$/+	-	-	9.9E-04	6,8E-08	1.7E-01
batman$^{l(2)k07907}$/+	-	-	3.8E-05	-	1.1E-02
batman$^{l(2)k02512}$/ *batman*$^{l(2)k07907}$	9.9E-04	3.8E-05	-	-	5.8E-02

Table S1B' : ΔON (%)

	Canton-S
batman$^{l(2)k02512}$/+	19.0
batman$^{l(2)k07907}$/+	14.1
batman$^{l(2)k02512}$ / *batman*$^{l(2)k07907}$	30.7
p10 batman$^{l(2)k02512}$ / +	5.9
p10 batman$^{l(2)k02512}$ / *batman*$^{l(2)k07907}$	23.4

Table S1C : p-values (p)

	psq^{0115}/+	*batman*$^{l(2)k02512}$/+	*Trl*^{13c}/ +
batman$^{l(2)k02512}$/ *psq*^{0115}	2,0E-10	1,1E-11	-
batman$^{l(2)k02512}$/+ ; *Trl*^{13c}/+	-	4,3E-01	5,8E-03

Table S1D : p-values (p)

	Canton-S	*psq*^{0115}/+	*Trl*$^{81.1}$/+	*batman*$^{l(2)k02512}$/+	*psq*^{0115}/+ ; *Trl*$^{81.1}$/+	*batman*$^{l(2)k02512}$/+ ; *Trl*$^{81.1}$/+
psq^{0115}/+	1.3E-02	-	-	-	-	-
Trl$^{81.1}$/+	5.0E-07	-	-	-	-	-
batman$^{l(2)k02512}$/+	3.1E-03	-	-	-	-	-
psq^{0115}/+ ; *Trl*$^{81.1}$/+	1.3E-10	8.5E-04	2.1E-05	-	-	-
batman$^{l(2)k02512}$/+ ; *Trl*$^{81.1}$/+	3.9E-04	-	2.8E-01	2.1E-01	-	-
batman$^{l(2)k02512}$/*psq*^{0115} ; *Trl*$^{81.1}$/+	4.7E-11	-	-	-	3.1E-01	8.0E-04

Table S1D' : ΔON (%)

	Canton-S
psq^{0115}/+	11.1
Trl$^{81.1}$/+	19.0
batman$^{l(2)k02512}$/+	10.6
psq^{0115}/+ ; *Trl*$^{81.1}$/+	31.7
batman$^{l(2)k02512}$/+ ; *Trl*$^{81.1}$/+	14.8
batman$^{l(2)k02512}$/*psq*^{0115} ; *Trl*$^{81.1}$/+	28
