# Supplementary material for: Genetic Basis for Developmental Homeostasis of Germline Stem Cell Niche Number: A Network of Tramtrack-Group Nuclear BTB Factors
Source: PLoS One. 2012 Nov 21;7(11):e49958. doi: 10.1371/journal.pone.0049958 (PMC3503823; doi:10.1371/journal.pone.0049958)
Supplement: Table S2 — Statistical significance of comparisons between the mean number of ovarioles in bab>psqIR flies and in control flies. (PDF) [file pone.0049958.s004.pdf]

Table S2. Statistical significance of comparisons between the mean number of ovarioles in bab>psqIR flies and in control flies.
Data from Figure 2.
S2A: p-value (p) was calculated using Student’s t-test to compare mean values of *bab>psqIR* samples and controls. (red/green code as in Table S1)
S2B: ∆ON (in %) was calculated for *bab>psqIR* relative to Canton-S, the bab-Gal4 driver alone (*bab*>), and the UAS-psqIR RNAi line alone (>*psqIR*) .

Table S2A : p-values (p)

	Canton-S	*bab>*	*>psqIR*
bab>psqIR	7.4E-03	1.6E-03	4.0E-03

Table S2B : ΔON (%)

	Canton-S	*bab>*	*>psqIR*
bab>psqIR	8.3	11.0	9.4
