# Supplementary material for: Genetic Basis for Developmental Homeostasis of Germline Stem Cell Niche Number: A Network of Tramtrack-Group Nuclear BTB Factors
Source: PLoS One. 2012 Nov 21;7(11):e49958. doi: 10.1371/journal.pone.0049958 (PMC3503823; doi:10.1371/journal.pone.0049958)
Supplement: Table S3 — Statistical significance of comparisons between the mean number of ovarioles in combinations of bab and other ttk-BTB group mutations. (PDF) [file pone.0049958.s005.pdf]

Table S3 Statistical significance of comparisons between the mean number of ovarioles in combinations of *bab* and other ttk-BTB group mutations.

Data from Figure 3.
S3A-B: p-value (p) was calculated using Student's t-test between samples of appropriate genotypes.
S3A'-B': ∆ON (in %) was calculated relative to Canton-S.

Table S3A : p-values (p)

	bab^{P}/+	bab^{pr72}/+	bab^{E1}/+	bab^{AR07}/+
Canton-S	2.6E-06	3.0E-04	3.6E-09	4.4E-03

Table S3A' : ∆ON (%)

	Canton-S
bab^{P}/+	16.1
bab^{pr72}/+	12.5
bab^{E1}/+	18.8
bab^{AR07}/+	7.8

Table S3B : p-values (p)

	Canton-S	bab^{P}/+	$ban^{l(2)k02512}$/+	$Trl^{81.1}$/+	psq^{0115}/+
Canton-S	-	3.8E-06	3.1E-03	5.0E-07	4.7E-02
$ban^{l(2)k02512}$/+; bab^{P}/+	6.5E-04	1.8E-01	3.0E-01	-	-
$Trl^{81.1}$/bab^{P}	3.7E-11	8.1E-04	-	1.9E-04	-
psq^{0115}/+ ; bab^{P}/+	1.0E-15	1.0E-07	-	-	4.3E-09

Table S3B' : ∆ON (%)

	Canton-S
bab^{P}/+	19.8
$ban^{l(2)k02512}$/+	10.6
$Trl^{81.1}$/+	19.0
psq^{0115}/+	9.5
$ban^{l(2)k02512}$/+; bab^{P}/+	14.3
$Trl^{81.1}$/bab^{P}	34.4
psq^{0115}/+ ; bab^{P}/+	43.9
