# Supplementary material for: Genetic Basis for Developmental Homeostasis of Germline Stem Cell Niche Number: A Network of Tramtrack-Group Nuclear BTB Factors
Source: PLoS One. 2012 Nov 21;7(11):e49958. doi: 10.1371/journal.pone.0049958 (PMC3503823; doi:10.1371/journal.pone.0049958)
Supplement: Table S4 — Statistical significance for comparisons of cellular parameters between pupal or adult ovaries between heterozygous combinations of bab and psq mutations. (PDF) [file pone.0049958.s006.pdf]

Table S4. Statistical significance for comparisons of cellular parameters between pupal or adult ovaries between heterozygous combinations of *bab* and *psq* mutations

Data from Figure 4.
p-value (p) was calculated using Student's t-test between samples of appropriate genotypes. Color code as in Table S1.

Table S4A Pupa : p-values (p) for TFC number per TF

	$bab^{P}/+$	$psq^{0115}/+$	$psq^{0115}/+ ; bab^{P}/+$
Canton-S	1.0E-07	3.4E-01	1.7E-13
$psq^{0115}/+ ; bab^{P}/+$	8.1E-02	1.7E-12	-

Table S4B Pupa : p-values (p) for TFC volume

	$bab^{P}/+$	$psq^{0115}/+$
Canton-S	6.4E-03	4.4E-01

Table S4C Pupa : p-values (p) for TFC number per ovary

	$bab^{P}/+$	$psq^{0115}/+$	$psq^{0115}/+ ; bab^{P}/+$
Canton-S	7.2E-01	1.9E-02	1.7E-04
$psq^{0115}/+ ; bab^{P}/+$	1.8E-03	2.5E-02	-

Table S4D Adult : p-values (p) for TFC number per TF

	$bab^{P}/+$	$psq^{0115}/+$	$psq^{0115}/+ ; bab^{P}/+$
Canton-S	8.6E-10	7.8E-01	2.2E-21
$psq^{0115}/+ ; bab^{P}/+$	5.5E-06	1.1E-26	-

Table S4E Adult : p-values (p) for CC number per TF

	$bab^{P}/+$	$psq^{0115}/+$
Canton-S	2.7E-01	5.4E-01
