# Supplementary material for: Genetic Basis for Developmental Homeostasis of Germline Stem Cell Niche Number: A Network of Tramtrack-Group Nuclear BTB Factors
Source: PLoS One. 2012 Nov 21;7(11):e49958. doi: 10.1371/journal.pone.0049958 (PMC3503823; doi:10.1371/journal.pone.0049958)
Supplement: Table S5 — (PDF) [file pone.0049958.s007.pdf]

Table S5A. TF number per ovary at the pupal stage

	Canton-S	*bab*^{P}/+	*psq*^{0115}/+	*psq*^{0115}/+ ; *bab*^{P}/+
Mean	21.22	24.60	24.57	31.83
Standard Deviation	0.74	1.17	0.95	0.13

Table S5B. Difference in the mean TF number between wild type, simple and double heterozygotes at the pupal stage

	bab^{P}/+	*psq*^{0115}/+	*psq*^{0115}/+ ; *bab*^{P}/+
Canton-S	3.38	3.35	10.61
bab^{P}/+	-	0.03	7.23
psq^{0115}/+	-	-	7.26
